# Histogram-based features track Alzheimer's progression in brain MRI

**DOI:** 10.1038/s41598-023-50631-1

**Published:** 2024-01-02

**Authors:** Nikaash Pasnoori, Thania Flores-Garcia, Buket D. Barkana

**Affiliations:** 1 Biomedical Engineering Department, University of Bridgeport, Bridgeport, CT 06604 USA; 2 Computer Engineering Department, University of Bridgeport, Bridgeport, CT 06604 USA; 3 Biomedical Engineering Department, The University of Akron, Akron, OH 44325-0302 USA

**Keywords:** Diseases, Health care, Engineering

## Abstract

Alzheimer's disease is a form of general dementia marked by amyloid plaques, neurofibrillary tangles, and neuron degeneration. The disease has no cure, and early detection is critical in improving patient outcomes. Magnetic resonance imaging (MRI) is important in measuring neurodegeneration during the disease. Computer-aided image processing tools have been used to aid medical professionals in ascertaining a diagnosis of Alzheimer's in its early stages. As characteristics of non and very-mild dementia stages overlap, tracking the progression is challenging. Our work developed an adaptive multi-thresholding algorithm based on the morphology of the smoothed histogram to define features identifying neurodegeneration and track its progression as non, very mild, mild, and moderate. Gray and white matter volume, statistical moments, multi-thresholds, shrinkage, gray-to-white matter ratio, and three distance and angle values are mathematically derived. Decision tree, discriminant analysis, Naïve Bayes, SVM, KNN, ensemble, and neural network classifiers are designed to evaluate the proposed methodology with the performance metrics accuracy, recall, specificity, precision, F1 score, Matthew’s correlation coefficient, and Kappa values. Experimental results showed that the proposed features successfully label the neurodegeneration stages.

## Introduction

**Figure 1:**
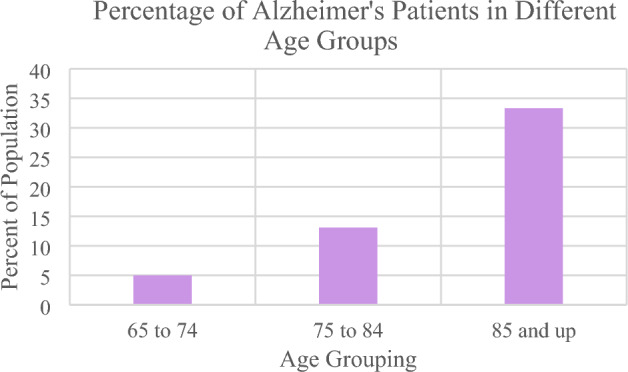
This graph shows the increasing incidence of Alzheimer's with age^1^.

Alzheimer's disease (AD) is a specific form of dementia noted for the widespread deterioration of neural tissue it causes. This degenerative disease primarily affects elderly individuals, with a high co-morbidity among patients over 60^1^. As the patient ages, the risk of developing the disease increases exponentially. For example, between the ages of 60 and 85, there is a 15-fold increase in disease prevalence, as seen in Fig. 1. Once the onset of the disease begins, treatment only focuses on ameliorating symptoms. No current cure is available^2^. As such, acceptable patient outcomes depend on early detection of the disease. Dementia is a broad term that describes a decline in cognitive skills and memory over a long period. This decline is so pronounced that it affects day-to-day living. While there are many forms of dementia, AD is the most common— it is identified as the primary cause in 70% of cases^3^. As of 2020, 50 million cases of Alzheimer's have been identified. The burden on the healthcare system is significant—over $1 trillion is spent globally mitigating the disease^4^. This cost is due to the substantial post-diagnosis care required for Alzheimer's patients. Regarding global prevalence, AD is the most common in the United States. This can be linked to increased surveillance and advanced detection methods in this country^5^.

Several clinical signs mark the pathophysiology of AD, including neuronal degeneration. Neural degeneration is another pathological hallmark of AD. As the disease progresses, the white and grey matter of the brain deteriorates as neurons die^6^. This clinical sign is especially important as it can be detected via Magnetic Resonance Imaging (MRI) by clinical professionals.

**Figure 2:**
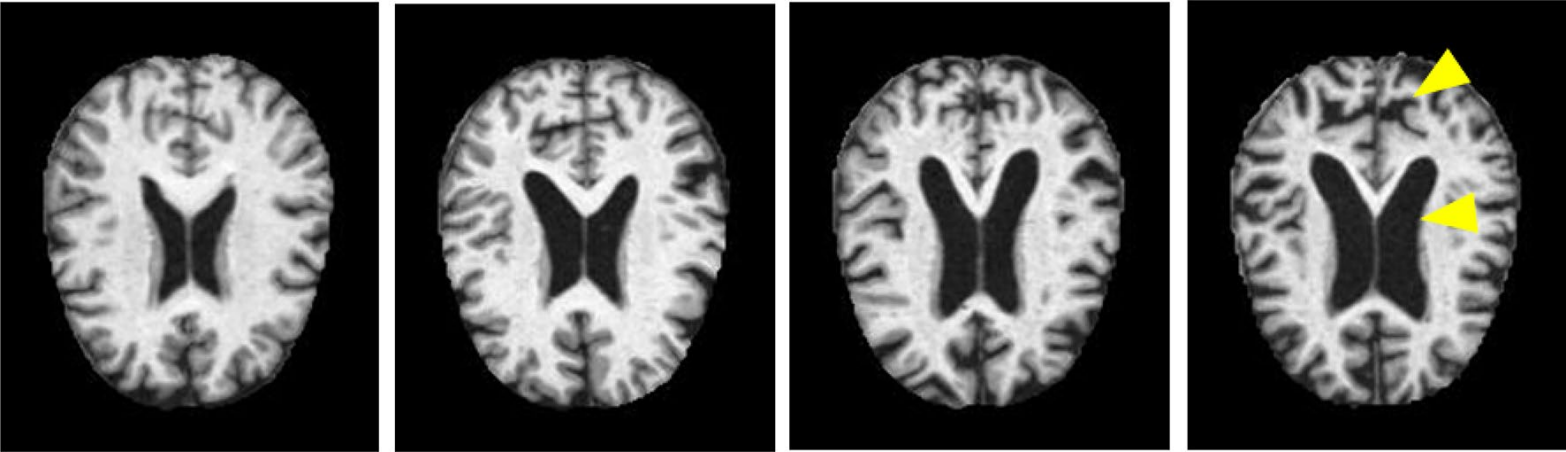
From left to right, a brain with no Alzheimer's, a brain with very mild symptoms, a brain with mild symptoms, and a brain with moderate symptoms. The yellow arrows in the moderate symptom image show the enlarged folds in the brain tissue and ventricles.

One of the key signs of the disease is the changing of tissue volumes in the brain over time. As AD progresses, neural tissue atrophy becomes more and more notable^7^. This progression can be seen in Fig. 2. A clinician can observe these changes in a patient over time and make a positive or negative diagnosis of Alzheimer's based on the atrophy^8^. To perform this procedure, MRI scans are the preferred method of diagnosis due to their non-invasive nature.

Building off previous algorithmic models for AD detection, we hand-crafted features based on an adaptive multi-thresholding algorithm to track AD progression, which ranges from no AD to moderate advancement.

## Methodology

**Figure 3:**
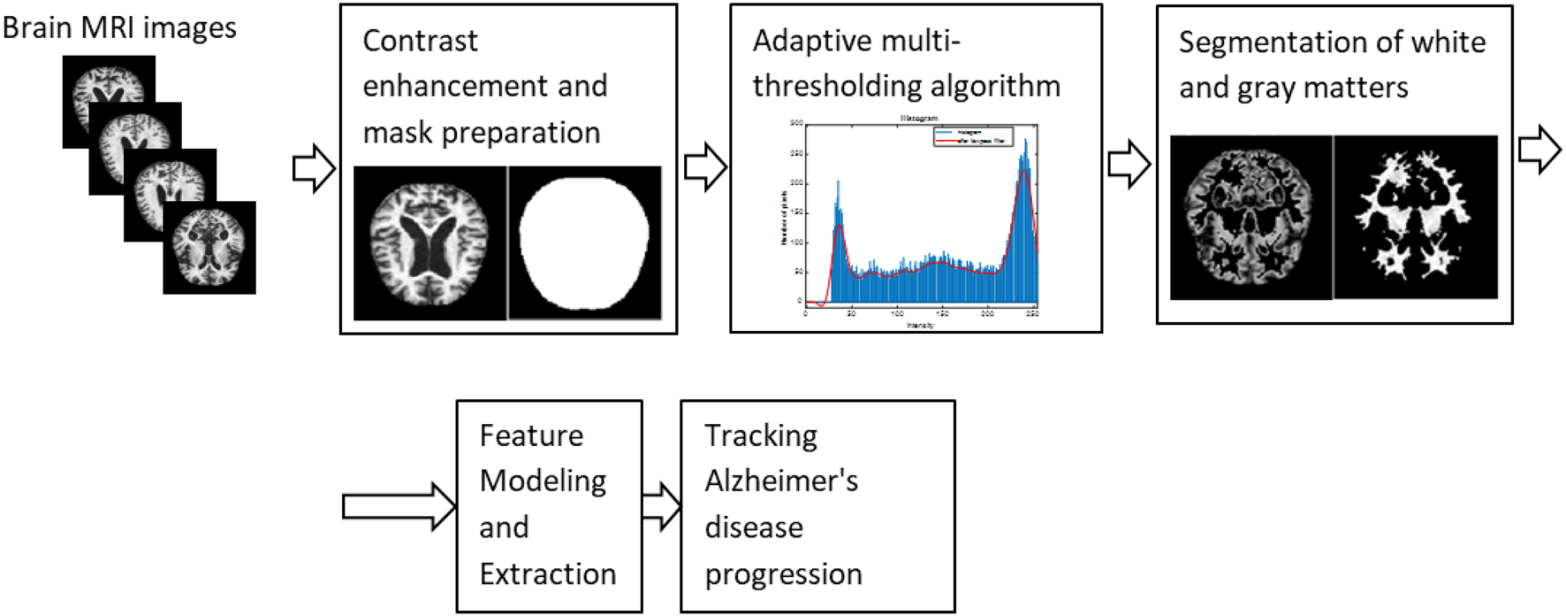
The outline of the proposed AD progression tracking model.

The model has the following steps: contrast enhancement, adaptive thresholding algorithm, white and gray matter segmentation, feature modeling and extraction, and machine learning models for tracking AD progression. Figure 3 depicts the mentioned stages.

### Contrast enhancement and mask preparation

The contrast between the white and gray matter in brain MRI images is often low, so segmentation of the tissues becomes difficult. Increasing contrast between the two matters is needed to analyze dementia stages accurately. We used linear stretching to prevent any gray-and-white matter ratio change. Linear stretching maps the minimum intensity value to 0 and the maximum intensity value to the highest intensity value, which is 255 in our image dataset. It linearly scales the values in between.

Consider an image $I\left(m,n\right)$ of size $M\times N$ pixels. ${I}_{C}\left(m,n\right)$ is the contrast-enhanced image obtained using linear stretching. ${I}_{min}$ and ${I}_{max}$ are the minimum and maximum intensity values in the image and k is the number of bits, which is 8 in the employed dataset.

1 $${I}_{C}\left(m,n\right)=\frac{I\left(m,n\right)-{I}_{min}}{{I}_{max}- {I}_{min}}\times ({2}^{k}-1)$$

**Figure 4:**
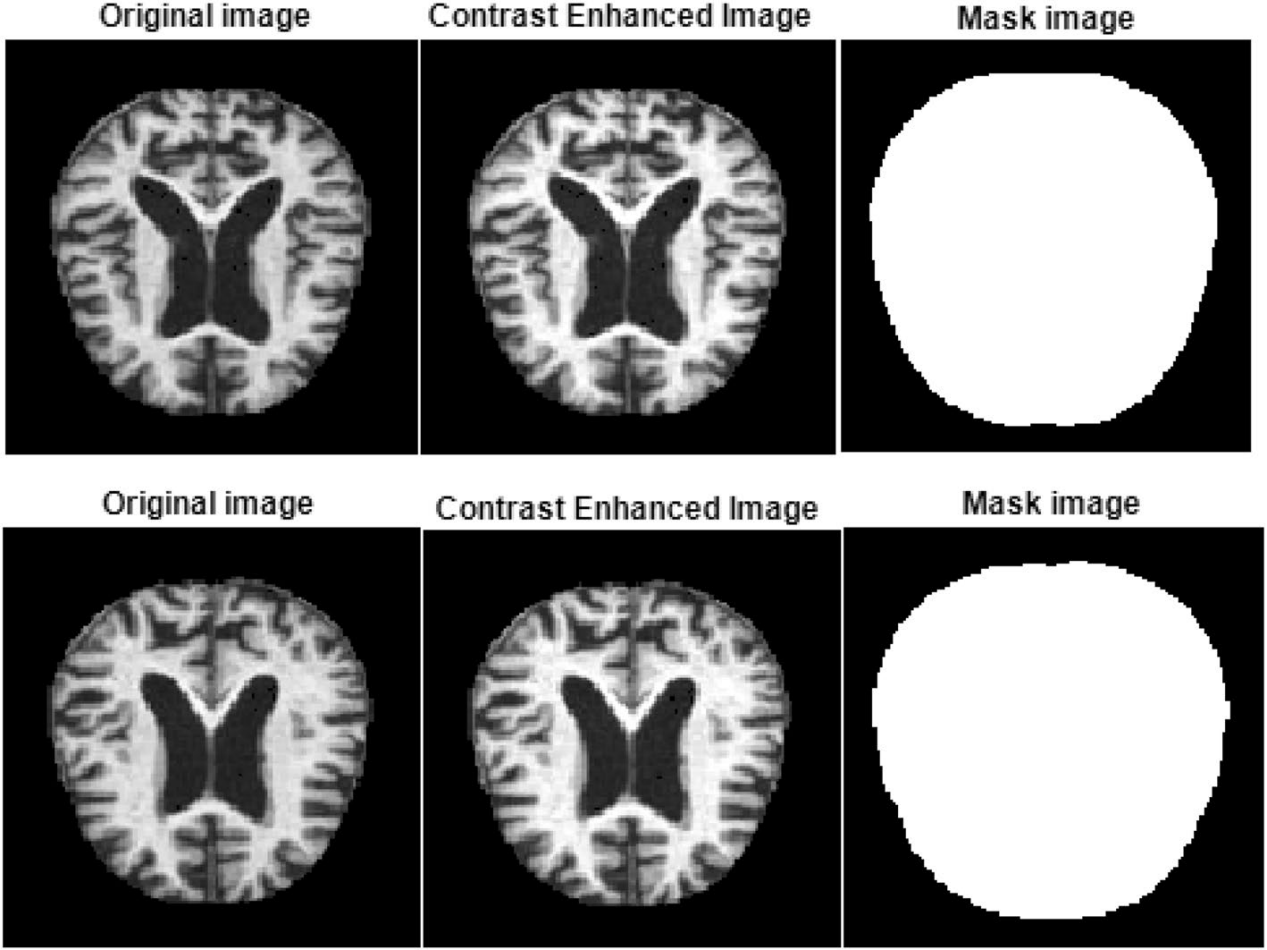
Contrast enhancement and masking showing two moderate dementia cases.

Figure 4 shows the results for non-dementia and moderate dementia cases. This step also standardizes the variety of image intensities.

### Adaptive multi-thresholding algorithm

**Figure 5:**
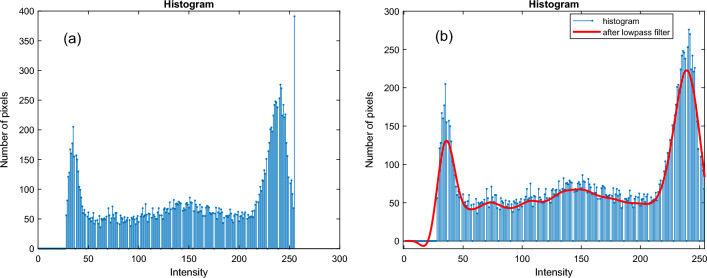
(**a**) Histogram of a non-dementia case, (**b**) Red line shows the smoothed histogram by the low pass filter.

Measuring the grey-to-white matter ratio (GWR) requires segmenting the grey and white matter. We developed an adaptive multi-thresholding algorithm to calculate two threshold values from the histogram of contrast-enhanced images. Figure 5a depicts the histogram of one of the non-dementia cases. A lowpass filter with a 0.005 rad/s normalized frequency was applied to the histogram to smooth the envelope of the histogram; see Fig. 5b. The lowpass filter uses a minimum-order filter with a stopband attenuation of 60 dB and compensates for the delay introduced by the filter. Two scalar threshold values are determined by using the smoothed envelope. Note that the lowpass filter does not affect the high-intensity information in the MRI images.

**Figure 6:**
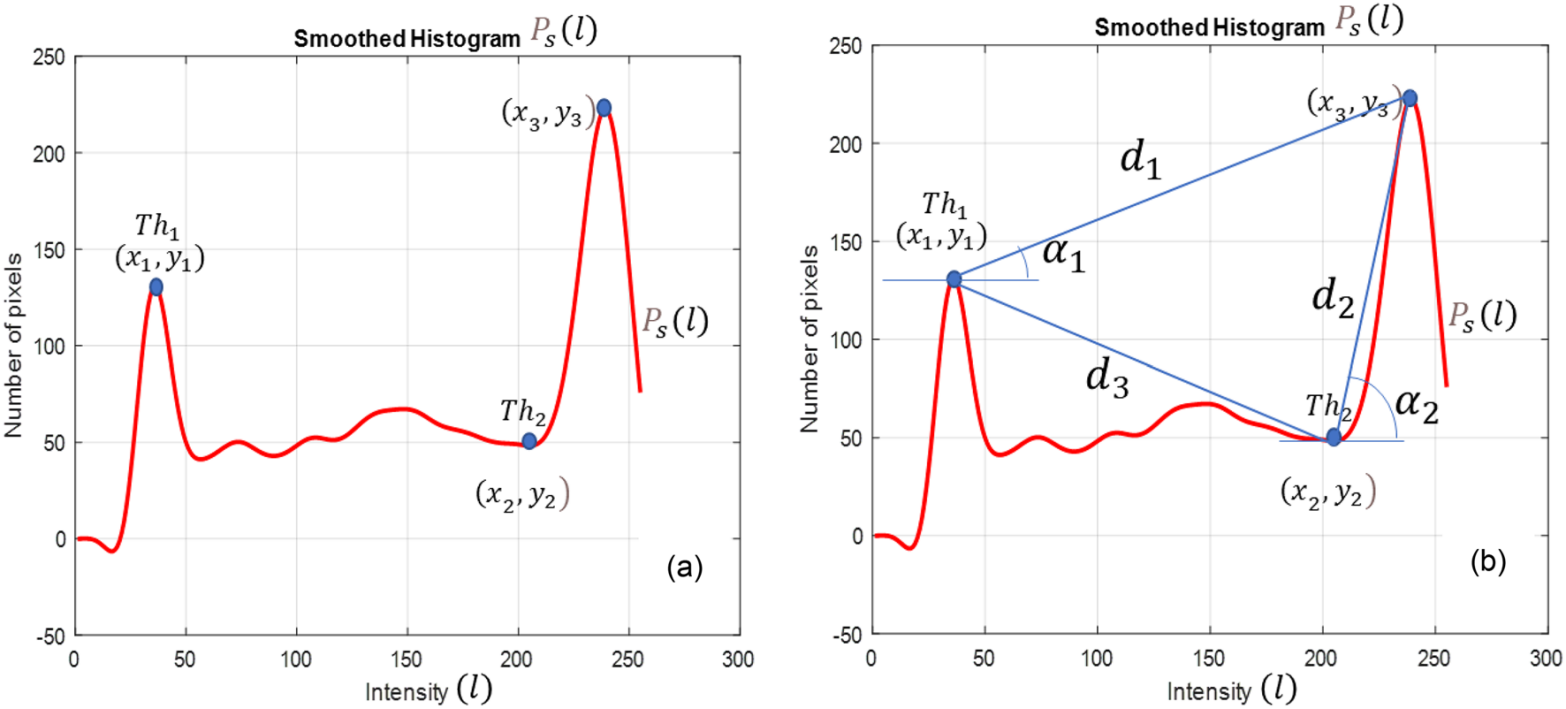
(**a**) Two thresholding values $T{h}_{1}$ and $T{h}_{2}$ are shown on the smoothed histogram ${P}_{s}(l)$. $T{h}_{1}$ is defined as the first maxima and $T{h}_{2}$ is the last minima of the function. (**b**) Two slopes.

Two thresholding values were calculated using the smoothed histogram. Figure 6a and b depict the adaptive multi-thresholding algorithm and measured features using it.

${\alpha }_{1}$ and ${\alpha }_{2}$ are defined —three distance parameters ${d}_{1}$, ${d}_{2}$, ${d}_{3}$ are defined.

${P}_{{I}_{c}}\left(l\right)$ is the histogram of the ${I}_{C}\left(m,n\right)$ and calculated as below.

2 $${P}_{{I}_{c}}\left(l\right)=\sum_{m=0}^{M-1}\sum_{n=0}^{N-1}{\delta }_{d}\left[{I}_{c}\left(m,n\right)-l\right]$$

where $l=0, 1, 2, \dots , L-1$ and delta function $${\delta }_{d}[k]=\left\{\begin{array}{c}1 \,if\, k=0\\ 0 \,if\, k\ne 0\end{array}\right.$$. The sum of all the entries in a histogram equals the total number of pixels in the image, $\sum_{l=0}^{L-1}{P}_{lc}(l)=MN.$^9^. The smoothed histogram ${P}_{s}$ was obtained using Eq. (2), ${H}_{L}$ is the low pass filter as $*$ represents the convolution operation.

3 $${P}_{s}={H}_{L}*{P}_{{I}_{c}}$$

Threshold points $\left({x}_{1}, {y}_{1}\right), \left({x}_{2}, {y}_{2}\right)$ are the local minimum values of ${P}_{s}$. The coordinates $\left({x}_{1}, {y}_{1}\right)$ and $\left({x}_{2}, {y}_{2}\right)$ are the second and the last local minimum on the x-axis. Thresholds are defined as

4 $$T{h}_{1}={x}_{1} \,and\, T{h}_{2}={x}_{2}$$

$\left({x}_{3}, {y}_{3}\right)$ is the absolute maximum of ${P}_{s}$. This point is used to calculate the slopes and distances between the threshold points. We defined the following parameters in Eqs. (5) through (13) to investigate the possibility of their use in the analysis of different dementia stages.

5 $${\mathrm{\alpha }}_{1}=\frac{{{\text{y}}}_{3}-{{\text{y}}}_{1}}{{{\text{x}}}_{3}-{{\text{x}}}_{1}}$$

6 $${\alpha }_{2}=\frac{{y}_{3}-{y}_{2}}{{x}_{3}-{x}_{2}}$$

7 $${d}_{1}=\sqrt{{\left({x}_{3}-{x}_{1}\right)}^{2}+{\left({y}_{3}-{y}_{1}\right)}^{2}}$$

8 $${d}_{2}=\sqrt{{\left({x}_{3}-{x}_{2}\right)}^{2}+{\left({y}_{3}-{y}_{2}\right)}^{2}}$$

9 $${d}_{3}=\sqrt{{\left({x}_{2}-{x}_{1}\right)}^{2}+{\left({y}_{2}-{y}_{1}\right)}^{2}}.$$

**Figure 7:**
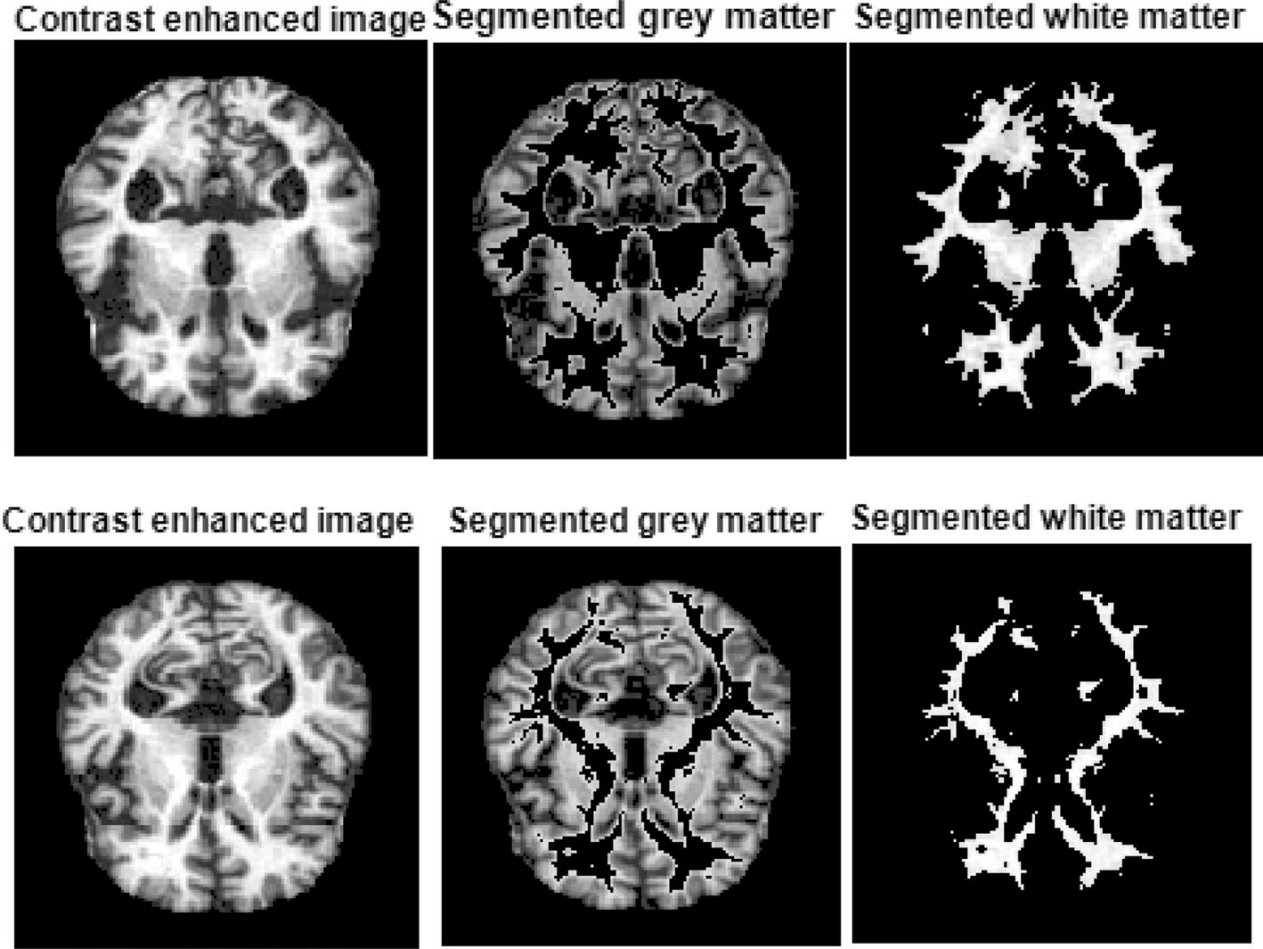
Segmented grey and white matters using the $T{h}_{1}$ and $T{h}_{2}$ values. Both cases represent moderate dementia.

The grey and white matter segmentation is performed by using the threshold values. In the equations below, ${I}_{gm}\left(m,n\right)$ and ${I}_{wm}\left(m,n\right)$ represent the grey and white matters, respectively. Volume, mean, and standard deviation of the grey and white matter are calculated and employed as potential features. GWR is the grey-to-white matter ratio calculated by Eqs. (10) and (11). Figure 7 shows the segmented white and gray matter obtained by using the $T{h}_{1}$ and $T{h}_{2}$ values.

10 $${I}_{gm}\left(m,n\right)=I\left(m,n\right)>T{h}_{1} AND I\left(m,n\right)<T{h}_{2}$$

11 $${I}_{wm}\left(m,n\right)=I\left(m,n\right)>T{h}_{2}$$

12 $$GWR=\frac{\sum_{m=0}^{M-1}\sum_{n=0}^{N-1}{I}_{gm}\left(m,n\right)}{\sum_{m=0}^{M-1}\sum_{n=0}^{N-1}{I}_{wm}\left(m,n\right)}$$

13 $$srinkage=\frac{\sum_{m=0}^{M-1}\sum_{n=0}^{N-1}{I}_{gm}\left(m,n\right)+\sum_{m=0}^{M-1}\sum_{n=0}^{N-1}{I}_{wm}\left(m,n\right)}{\sum_{m=0}^{M-1}\sum_{n=0}^{N-1}{I}_{mask}\left(m,n\right)}$$

## Database

**Figure 8:**
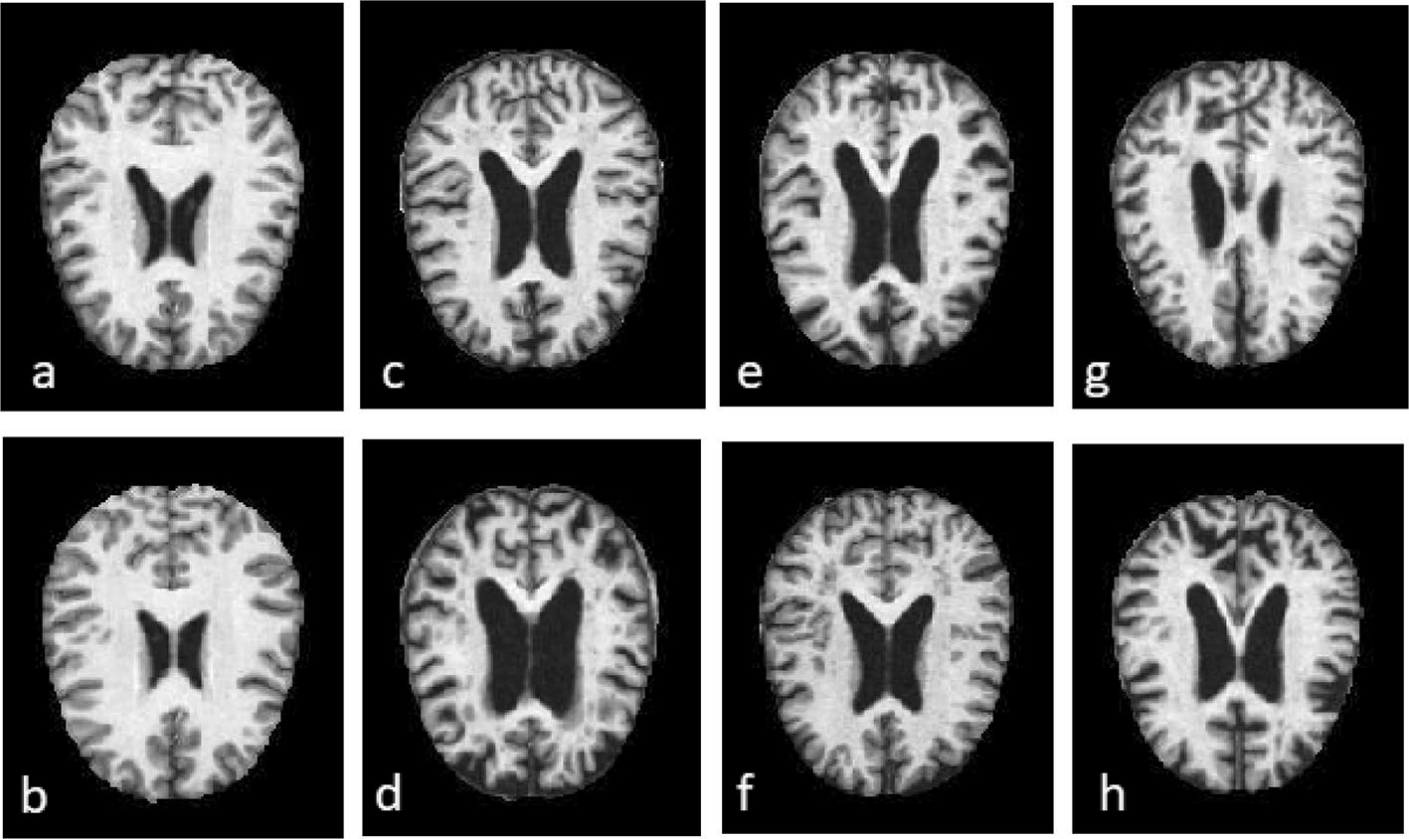
MRI images of dementia cases (**a**,**b**) Non-dementia, (**c**,**d**) Very mild dementia, (**e**,**f**) Mild dementia, and (**g**,**h**) Moderate dementia.

Alzheimer's disease MRI images were obtained from Kaggle, an open-source website^10^. For 4-class classification studies, the dataset contains MRI images of four stages of the disease as non-demented (3200 images), very mild demented (2240 images), mild demented (897 images), and moderate demented (64 images). The set is imbalanced. The image resolution is 128 $\times$ 128. Binary classification studies merge very mild, mild, and moderate classes. In that case, the set has 3200 images for both classes. Some of these images can be seen in Fig. 8. The set is known as the Kaggle set and is widely used in AD research for evaluating models.

## Experimental results and analysis

**Figure 9:**
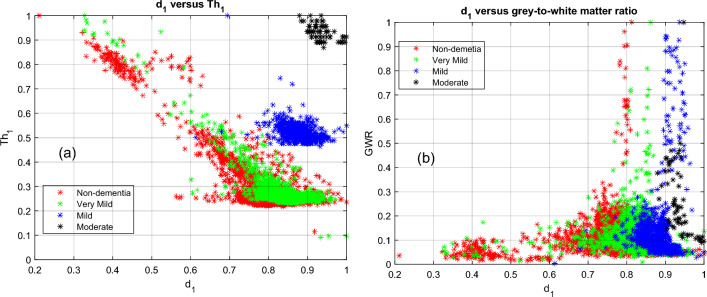
Non-dementia, very mild, mild, and moderate dementia classes. a) ${d}_{1}$ versus $T{h}_{1}$; b) ${d}_{1}$ versus grey-to-white matter ratio (GWR).

The reduction in pixel intensity can be explained by tissue atrophy in the brain as Alzheimer's progresses. As grey matter atrophies, it is replaced by void space, which appears as black pixels in an MRI^11^. Alzheimer's deterioration occurs within grey matter first, as it is the external tissue of the brain. Therefore, the grey matter tissue volume will decrease before the white matter does. The calculated features are analyzed in terms of their efficiency in labeling the dementia stages. Figures 9 through 11 show the 2D and 3D plots of four dementia stages for measured features.

**Figure 10:**
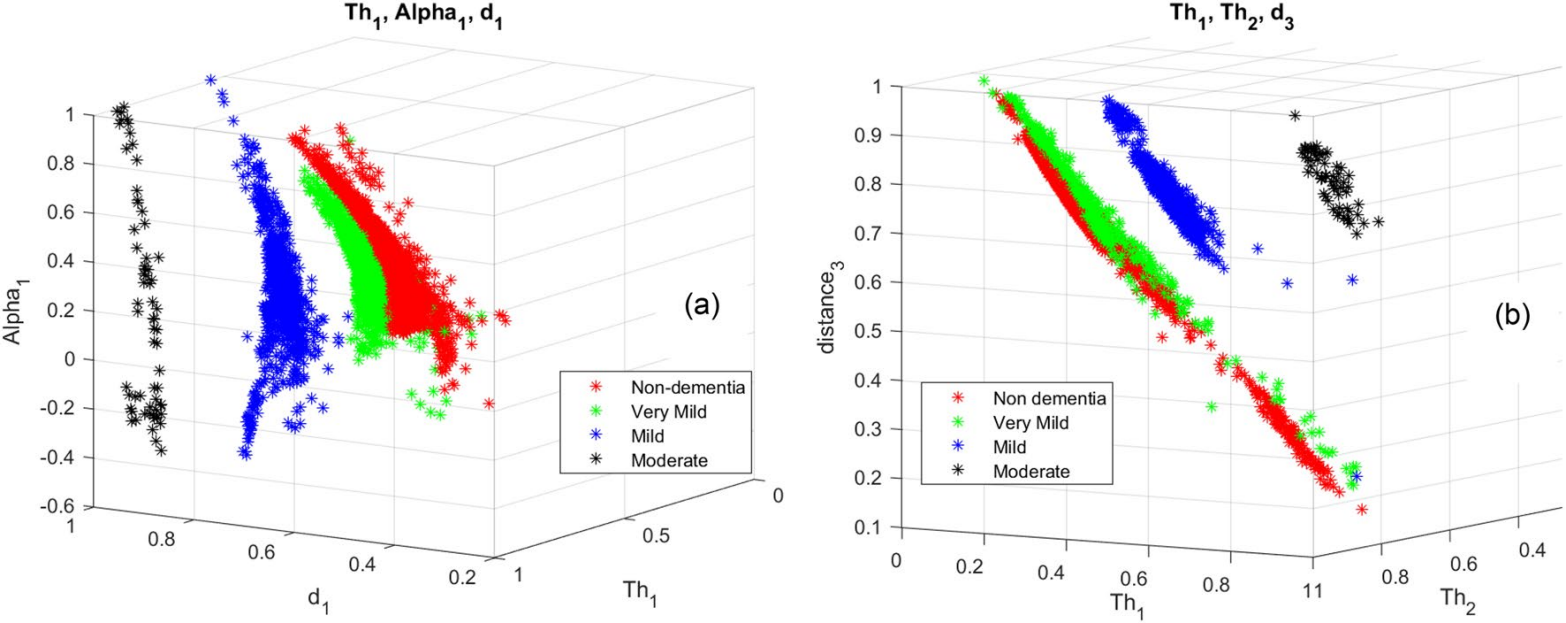
(**a**) $T{h}_{1}$, α1, and ${d}_{1}$ plot, (**b**) $T{h}_{1}$, $T{h}_{2}$, and ${d}_{3}$ plot of non-dementia, very mild, mild, and moderate dementia classes.

**Figure 11:**
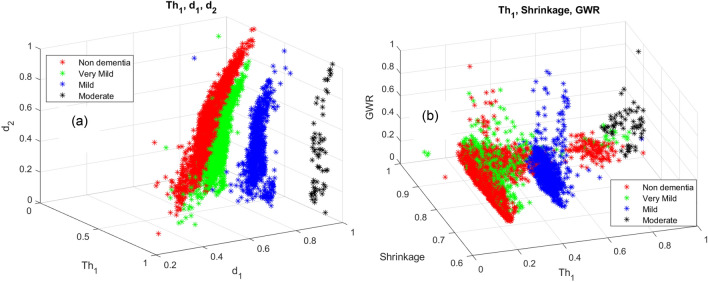
(**a**) $T{h}_{1}$, ${d}_{1}$, and ${d}_{2}$. (**b**) $T{h}_{1}$, shrinkage, and GWR plot of non-dementia, very mild, mild, and moderate dementia classes.

**Figure 12:**
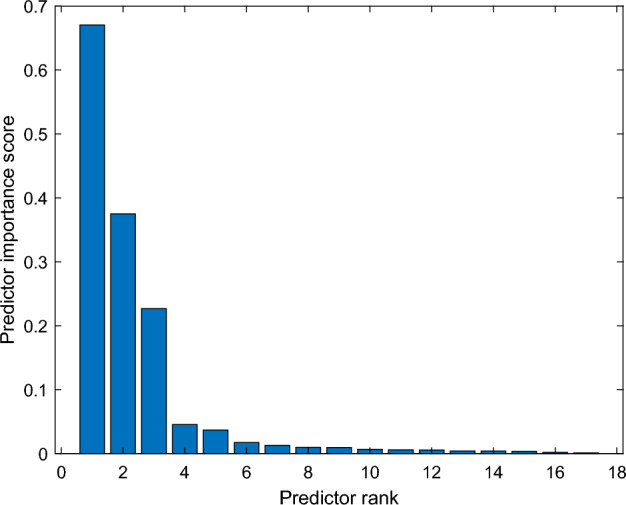
Feature ranking using minimum redundancy maximum relevance (MRMR) algorithm.

It is observed that $T{h}_{1}$ and ${d}_{1}$ features are effective in distinguishing moderate and mild dementia. However, non-dementia and very mild dementia overlap significantly (shown in Fig. 9). Shrinkage, GWR, and especially $\mathrm{\alpha }1, { {\text{and}} d}_{2}$ features help distinguish non and very-mild dementia (shown in Figs. 10 and 11). We ranked the features using the minimum redundancy maximum relevance (MRMR) algorithm and calculated the most significant five features as $T{h}_{1}$, the mean intensity value of the white matter, ${d}_{1}$, shrinkage, and the volume of the white matter. The chi-square test univariate feature ranking algorithm supported the MRMR results. Figure 12 depicts the feature ranking using the MRMR algorithm. The drop in the importance score represents the confidence in the selection algorithm. There is a significant drop between the first, second, third, and fourth predictors, as seen in Fig. 12. The features after the fourth have a slight decrease in importance score referring to non-significant features.

**Figure 13:**
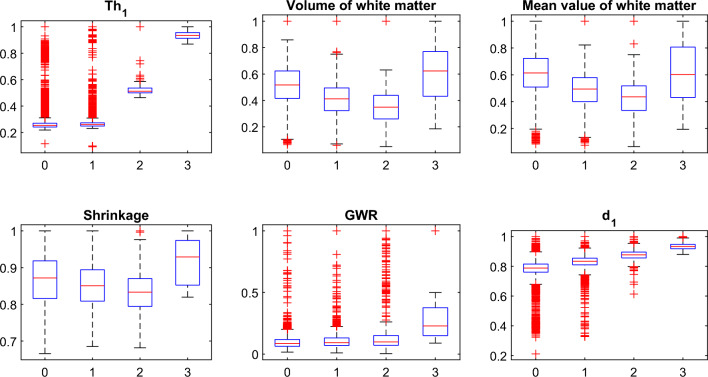
Statistical presentation of the most significant features in four dementia stages, coded as 0-non, 1-very mild, 2-mild, and 3-moderate dementia.

The statistical distribution of the most compelling features is given in Fig. 13. As can be seen, non and very-mild dementia have overlapping characteristics and more outliers compared to mild and moderate dementia. We observed that white matter characteristics are more effective in distinguishing different stages.

## Labeling dementia stages

We evaluated the performance of the defined features of AD in MRI images using decision trees, Discriminant Analysis, Naïve Bayes, Support vector machines (SVMs), KNN, Ensemble, and Neural Network (NN) classifiers. Appendix A presents the specifications of the designed classifiers, as Tables 1 and 2 show the performances. tenfold cross validation is employed with 60% of the dataset used for training, 20% for validation, and the remaining used for testing in each class. Model complexity is reduced by cross-validation to avoid overfitting. Table 1 uses all the features in labeling the AD stages, while Table 2 uses the most significant five features identified by the feature reduction algorithm in Sect. "Experimental results and analysis". Performance metrics are accuracy, recall, specificity, precision, F1 score, Matthews correlation coefficient, and Kappa values.

**Table 1:** Overall performances using all features, a total of 17, are employed.

Classifier	Kernel	Accuracy	Recall	Specificity	Precision	F1 Score	Matthews correlation coefficient	Kappa
Decision tree	Fine	0.97	0.98	0.99	0.98	0.98	0.97	0.92
Discriminant analysis	Quadratic	1.00	1.00	1.00	1.00	1.00	1.00	1.00
Naïve Bayes	Kernel	0.87	0.92	0.94	0.92	0.92	0.86	0.65
SVM	Quadratic	1.00	1.00	1.00	1.00	1.00	1.00	1.00
KNN	Fine	0.99	0.99	1.00	1.00	0.99	0.99	0.98
Ensemble	Subspace discriminant	1.00	1.00	1.00	1.00	1.00	1.00	1.00
Neural network	Narrow	1.00	1.00	1.00	1.00	1.00	1.00	1.00

**Table 2:** Overall performance of the most significant five features: $T{h}_{1}$, the mean intensity value of the white matter, ${d}_{1}$, shrinkage, and the volume of the white matter.

Classifier	Kernel	Accuracy	Recall	Specificity	Precision	F1 Score	Matthews correlation coefficient	Kappa
Decision Tree	Fine	0.94	0.96	0.97	0.96	0.96	0.93	0.83
Discriminant analysis	Quadratic	0.91	0.94	0.96	0.94	0.94	0.90	0.75
Naïve Bayes	Kernel	0.86	0.90	0.94	0.91	0.91	0.84	0.62
SVM	Quadratic	0.95	0.97	0.98	0.97	0.97	0.95	0.87
KNN	Fine	0.93	0.96	0.97	0.95	0.95	0.92	0.81
Ensemble	Subspace discriminant	0.84	0.90	0.93	0.91	0.90	0.83	0.58
Neural network	Narrow	0.96	0.97	0.98	0.98	0.98	0.96	0.89

Employing all features achieved higher performance than the five significant features. Discriminant analysis, SVM, Ensemble, and Neural network classifiers performed almost perfect classification. Tables 3 and 4 show the confusion matrices for narrow neural networks employing 17 (entire set) and 5 (the most significant) features. Classes are 0-non, 1-very mild, 2-mild, and 3-moderate dementia. Although the MRMR algorithm did not report the remaining features as effective, they perfected the classifier performances, as seen in Table 1. Figures 10 and 11 show mild and moderate dementia and minimize the overlap between non and very-mild dementia.

**Table 3:** Confusion matrix of Narrow Neural Network (NNN) from Table 1, employing 17 features.

		Predicted class			
		0	1	2	3
True class	0	3199	1	0	0
	1	0	2239	1	0
	2	0	1	895	0
	3	0	0	0	64

**Table 4:** Narrow Neural Network (NNN) from Table 2, employs 5 of the most significant features.

True class	Predicted class			
	0	1	2	3
0	3108	92	0	0
1	147	2092	1	0
2	1	2	892	1
3	0	0	0	64

Although the five features achieved high performance compared to the works in the literature, they failed to separate the non and very-mild dementia classes, which have overlapping features, observed in Fig. 13, a statistical presentation of the classes.

Table 5 presents the runtime and total cost metrics of the classifiers based on the11th Gen Intel(R) Core(TM) i7-11390H @ 3.40 GHz 2.92 GHz, 16.0 GB (15.7 GB usable), 64-bit operating system, × 64-based processor computer specifications. For the NNN classifier, the iteration limit was set to 1000.

**Table 5:** Runtime and total cost metrics of the classifiers.

Classifier	Kernel	Runtime all features (s)	Runtime five features (s)	Runtime average (s)	Total cost all features	Total cost five features	Average cost
Decision tree	Fine	2.66	0.97	1.82	193	367	280
Discriminant analysis	Quadratic	1.14	1	1.07	2	599	300.5
Naïve Bayes	Kernel	32.91	11.20	22.06	859	926	892.5
SVM	Quadratic	6.56	185.88	96.22	0	305	152.5
KNN	Fine	2.31	1.75	2.03	39	458	248.5
Ensemble	Subspace discriminant	3.30	2.59	2.95	5	1003	504
Neural network	Narrow	2.26	2.70	2.78	133	392	262.5

Feature extraction done automatically using convolutional neural networks (CNNs) or other deep neural networks (DNNs) has been trending in recent years. We compare the performance of the hand-crafted features extracted in this work with the other works in the literature in Table 6.

**Table 6:** Comparison of works in the literature using the Kaggle dataset for 4-class classification—overall performance metrics given in %.

Work	Year	Accuracy	Recall	Specificity	Precision	F1 score	Matthews correlation coefficient	Kappa
ADGNET	2020	99.61	99.69	99.53	99.53	99.61	–	99.22
Liang et al.^12^								
SimCLR	2020	93	93	94.70	93	93	–	87.70
Liang et al.^12^								
ResNetXt WSL	2020	93.53	94	93.18	91.26	92.61	–	86.87
Liang et al.^12^								
DEMNET	2021	95.23	95	-	96	95.27	–	93
(SMOTE)^13^								
DEMNET (without SMOTE)	2021	85	88	-	80	83	–	75
Murugan et al.^13^								
Kaplan et al.^14^	2021	99.62	99.66		99.74	99.65	–	–
Sharma et al.^15^	2022	94.92	94.94	98.30	94.93	–	–	–
Avsar and Polat^16^	2023	96.35	96	–	96	96	–	–
This work—NNN, (17 features)	2023	100	100	100	100	100	100	100
This work—NNN, (5 features)	2023	96	97	98	98	98	96	89

Liang and Gu proposed a weakly supervised learning (WSL)-based deep learning (DL) framework called ADGNET. It consisted of a backbone network, a task network, and image reconstruction. The work reported high performance, outperforming the state-of-the-art ResNeXt WSL and SimCLR models^12^. Murugan et al. designed a deep learning model DEMNET and evaluated it using the Kaggle dataset. DEMNET consists of a CNN to extract features using the normalized data. Their work achieved an overall accuracy of 95.23%, 95% of recall, and 96% of precision for 4-class classification^13^.

Kaplan et al. proposed a feed-forward local phase quantization network (LPQNet) consisting of multilevel feature generation, feature selection, and classification phases. The LPQNet was designed to have high accuracy and low computational complexity. The model was tested on a private AD dataset and the Kaggle dataset, achieving 99.62% accuracy on the Kaggle dataset using four classes^14^. In another study, Kaplan et al. used vision transformers and generated 16 exemplars. Several histogram-based feature extraction methods were used. Their work achieved 100% accuracy for binary classification using cubic support vector machine (CSVM) and fine KNN classifiers^17^. The shortfall of their work is that the details highlighting the healthy and AD slices were manually selected in MRI/CT images.

A 4-class AD detection model using CNN with activation Leaky ReLU was designed in Ref.^16^. The data was oversampled using SMOTE technique. They reported an overall accuracy of 96.35% on the Kaggle dataset. Sharma et al. used a transfer learning-based modified inception model, including normalization in the preprocessing stage for 4-class AD detection. Vertical and horizontal flipping, rotation, and brightness techniques were used in the augmentation step to balance the class sizes in the Kaggle dataset. Their work obtained 94.93%, 94.94%, 98.3%, and 94.92% precision, recall, specificity, and accuracy, respectively. Without any details, the authors stated that the work could not guarantee reproducibility^15^.

In another work, one machine-learning model took longitudinal brain scans and patient classifiers such as age to make informed decisions about the presence of AD. This model was able to predict cases of the disease with a 97.58% accuracy rating^11^. In work^18^, convolutional neural networks were used to detect AD patients from stable controls with an accuracy of 88%. Research has shown the grey-and-white matter ratio (GWR) decreases as the disease progresses^19^. The work in^20^ studied the changes in brain volume, focusing on the occipital lobe and hippocampal region. In a recent study, Agarwal et al. labeled cognitively normal, AD, and mild cognitive impairment using CNN-based DL models^21^ and achieved promising results. CCN-based feature extraction is done automatically by feeding the images to the model raw or after the preprocessing stage. The origin of the obtained features is not completely known. Considering that the brain has a very complicated structure and the etiology of AD is unknown, there is a need to develop mathematical descriptors to define the structure and changes in the brain. The advantage of our work is the hand-crafted feature modeling. The state-of-the-art works in the literature use CNN or similar deep-learning techniques to extract features to detect the AD stages. The performance of those models highly depends on the size of the dataset. Another work^22^ used hybrid clustering and a game theory-based approach to monitor the progress of AD using MRI images. Their work consisted of stages-registration, skull stripping, histogram normalization, feature selection, segmentation, and classification. Calculated features were based on the co-occurrence matrix. Evaluation of the work was limited to three performance metrics—accuracy, sensitivity, and specificity—and three previous studies. They reported accuracies between 83.45 and 88.83% with balanced sensitivity and specificity on the OASIS dataset. In our work, we mathematically modeled a set of features based on the histogram of the contrast enhanced MRI images that can be used to track Alzheimer's disease progression. In addition to employing supervised classifiers, unsupervised or rule-based classifiers can be designed using the proposed features.

## Conclusion

This work developed a model for tracking Alzheimer's disease progression. An adaptive multi-thresholding algorithm was proposed, and a set of novel features were mathematically defined. The system was evaluated on the Kaggle dataset consisting of non-demented, very mild, mild, and moderate dementia MRI images. The algorithm was based on the geometric shape of the envelope of the smoothed histogram. The grey and white matter were segmented using the obtained threshold values. The features included the grey-to-white matter ratio, shrinkage, slope values, white and grey matter volume, statistical moments, and distance parameters. The MRMR feature ranking algorithm, used for feature ranking and selection, showed high confidence for the five features containing $T{h}_{1}$, the mean intensity and volume of the white matter, ${d}_{1}$, and shrinkage.

The proposed model was evaluated by various classification algorithms—decision tree, discriminant analysis, Naïve Bayes, SVM, KNN, ensemble, and neural network classifiers. Performance metrics were accuracy, recall, specificity, precision, F1 score, Matthews correlation coefficient, and Kappa values. Discriminant analysis, SVM, ensemble, and neural network classifiers achieved perfect accuracy using all features.

### Supplementary Information

Supplementary Information.

## Data Availability

The work used an open-source dataset. Link https://www.kaggle.com/tourist55/alzheimers-dataset-4-class-of-images.
