# Supplementary material for: Histogram-based features track Alzheimer's progression in brain MRI
Source: Sci Rep. 2024 Jan 2;14:257. doi: 10.1038/s41598-023-50631-1 (PMC10761829; doi:10.1038/s41598-023-50631-1)
Supplement: Supplementary file 1 — Supplementary Information. [file 41598_2023_50631_MOESM1_ESM.docx]

| Appendix A. Designed classifier specifications.   \| Method \| Kernel \| Model Specification \| \| --- \| --- \| --- \| \| Decision Tree \| Fine \| Maximum number of splits: 100 Split criterion: Gini's diversity index Surrogate decision splits: Off \| \| Discriminant Analysis \| Quadratic \| Covariance structure: Full \| \| Naïve Bayes \| Kernel \| Distribution name for numeric predictors: Kernel Distribution name for categorical predictors: Not Applicable Kernel type: Gaussian Support: Unbounded \| \| SVM \| Quadratic \| Kernel function: Quadratic Kernel scale: Automatic Box constraint level: 1 Multiclass method: One-vs-One Standardize data: true \| \| KNN \| Fine \| Number of neighbors: 1 Distance metric: Euclidean Distance weight: Equal Standardize data: true \| \| Ensemble \| Subspace  Discriminant \| Ensemble method: Subspace Learner type: Discriminant Number of learners: 30 Subspace dimension: 9 \| \| NNN \| Narrow \| Number of fully connected layers: 1 First layer size: 10 Activation: ReLU Iteration limit: 1000 Regularization strength (Lambda): 0 Standardize data: Yes \| |  |  |  |
| --- | --- | --- | --- | --- | --- | --- | --- | --- | --- | --- | --- | --- | --- | --- | --- | --- | --- | --- | --- | --- | --- | --- | --- | --- | --- | --- | --- |
